# Long non-coding RNAs defining major subtypes of B cell precursor acute lymphoblastic leukemia

**DOI:** 10.1186/s13045-018-0692-3

**Published:** 2019-01-14

**Authors:** Alva Rani James, Michael P. Schroeder, Martin Neumann, Lorenz Bastian, Cornelia Eckert, Nicola Gökbuget, Jutta Ortiz Tanchez, Cornelia Schlee, Konstandina Isaakidis, Stefan Schwartz, Thomas Burmeister, Arend von Stackelberg, Michael A. Rieger, Stefanie Göllner, Martin Horstman, Martin Schrappe, Renate Kirschner-Schwabe, Monika Brüggemann, Carsten Müller-Tidow, Hubert Serve, Altuna Akalin, Claudia D. Baldus

**Affiliations:** 1 Department of Hematology and Oncology, Charité, University Hospital Berlin, Campus Benjamin Franklin, 12203 Berlin, Germany; 2 German Cancer Research Center (DKFZ), 69120 Heidelberg, Germany; 3 German Cancer Consortium (DKTK), 69120 Heidelberg, Germany; 4 Department of Pediatric Hematology/Oncology, Charité, University Hospital Berlin, Campus Rudolf Virchow, 13353 Berlin, Germany; 5 Department of Medicine II, Department of Hematology/Oncology, Goethe University Hospital, 60590 Frankfurt/M, Germany; 6 Department of Hematology, Oncology and Tumor Immunology, Charité University Hospital Berlin, Campus Virchow-Klinikum, 13353 Berlin, Germany; 7 Department of Hematology, Oncology & Rheumatology, University Clinic Heidelberg, 69120 Heidelberg, Germany; 8 Department of Pediatric Hematology and Oncology, Research Institute Children’s Cancer Center, University Medical Center Hamburg, 20251 Hamburg, Germany; 9 Department of Pediatrics, University Hospital Schleswig-Holstein, Campus Kiel, 24105 Kiel, Germany; 10 Department of Hematology and Oncology, University Hospital Schleswig-Holstein, Campus Kiel, 24105 Kiel, Germany; 11 Bioinformatics Platform, Berlin Institute for Medical Systems Biology (BIMSB), Max Delbrück Center (MDC), 13125 Berlin, Germany

**Keywords:** BCP-ALL subtypes, DUX4, Ph-like, NH-HeH, Subtype-specific lncRNAs, Key signaling pathways, Relapse-specific lncRNAs, Epigenetically altered lncRNAs

## Abstract

**Background:**

Long non-coding RNAs (lncRNAs) have emerged as a novel class of RNA due to its diverse mechanism in cancer development and progression. However, the role and expression pattern of lncRNAs in molecular subtypes of B cell acute lymphoblastic leukemia (BCP-ALL) have not yet been investigated. Here, we assess to what extent lncRNA expression and DNA methylation is driving the progression of relapsed BCP-ALL subtypes and we determine if the expression and DNA methylation profile of lncRNAs correlates with established BCP-ALL subtypes.

**Methods:**

We performed RNA sequencing and DNA methylation (Illumina Infinium microarray) of 40 diagnosis and 42 relapse samples from 45 BCP-ALL patients in a German cohort and quantified lncRNA expression. Unsupervised clustering was applied to ascertain and confirm that the lncRNA-based classification of the BCP-ALL molecular subtypes is present in both our cohort and an independent validation cohort of 47 patients. A differential expression and differential methylation analysis was applied to determine the subtype-specific, relapse-specific, and differentially methylated lncRNAs. Potential functions of subtype-specific lncRNAs were determined by using co-expression-based analysis on nearby (*cis*) and distally (*trans*) located protein-coding genes.

**Results:**

Using an integrative Bioinformatics analysis, we developed a comprehensive catalog of 1235 aberrantly dysregulated BCP-ALL subtype-specific and 942 relapse-specific lncRNAs and the methylation profile of three subtypes of BCP-ALL. The 1235 subtype-specific lncRNA signature represented a similar classification of the molecular subtypes of BCP-ALL in the independent validation cohort. We identified a strong correlation between the DUX4-specific lncRNAs and genes involved in the activation of TGF-β and Hippo signaling pathways. Similarly, Ph-like-specific lncRNAs were correlated with genes involved in the activation of PI3K-AKT, mTOR, and JAK-STAT signaling pathways. Interestingly, the relapse-specific lncRNAs correlated with the activation of metabolic and signaling pathways. Finally, we found 23 promoter methylated lncRNAs epigenetically facilitating their expression levels.

**Conclusion:**

Here, we describe a set of subtype-specific and relapse-specific lncRNAs from three major BCP-ALL subtypes and define their potential functions and epigenetic regulation. The subtype-specific lncRNAs are reproducible and can effectively stratify BCP-ALL subtypes. Our data uncover the diverse mechanism of action of lncRNAs in BCP-ALL subtypes defining which lncRNAs are involved in the pathogenesis of disease and are relevant for the stratification of BCP-ALL subtypes.

**Electronic supplementary material:**

The online version of this article (10.1186/s13045-018-0692-3) contains supplementary material, which is available to authorized users.

## Background

B cell precursor acute lymphoblastic leukemia (BCP-ALL) is the most prevalent disease in children but affects also adults. Despite improvements in treatment regimens such as chemotherapy and allogeneic hematopoietic stem cell transplantation, the prognosis remains poor for patients in high-risk groups and at relapse [1]. Various risk subtypes have been established based on the cytogenetic analysis and molecular genetics studies. These subtypes are classified based on the presence of high hyperdiploidy (51–65 chromosomes) [2], hypodiploidy (less than 44 chromosomes) [3], and fusion genes, such as BCR-ABL, ETV6-RUNX, and MLL [4]. About 70–80% of both adults and pediatric cases of BCP-ALL constitute these subtypes, although the frequency may differ [5].

Recent efforts taking advantage of whole transcriptome sequencing (RNA-seq) have refined this classification by identifying novel BCP-ALL subtypes [6]. RNA-seq analyses identified cytogenetically non-detectable recurrent rearrangements and gene fusions, which allowed characterization of additional subtypes based on distinct gene expression profiles [7]. For example, the DUX4 subtype is defined mainly by the IGH-DUX4 [8] gene fusions; the Ph-like subtype is a high-risk subtype with a gene expression profile similar to Ph-positive ALL, but lacking BCR-ABL1 fusion gene [9]; and the near haploid/high hyperdiploid (NH-HeH) (51–67 chromosomes) subtype is a common subtype [10, 11] comprising 30% of all pediatric BCP-ALL. These subtypes are clinically relevant with distinct gene expression profile and have been widely studied in the recent past.

Nevertheless, we are far from complete understanding of BCP-ALL subtypes and their heterogeneity and its associated molecular mechanisms, which pose a major challenge for improving diagnosis and therapy. Recent studies have suggested that long non-coding RNAs (lncRNAs) and small non-coding RNAs (e.g., microRNAs) play a key role in development and progression of leukemia [11] and thus constitute as new biomarkers and potential targets for novel therapies [12].

lncRNAs are arbitrarily defined as transcripts longer than 200 bp and lacking an extended protein-coding open reading frame (ORF). It has become apparent that lncRNAs are frequently spliced and polyadenylated and are mainly transcribed by RNA polymerase II [13]. lncRNA expression has been reported as highly tissue-specific even though the expression abundance is generally lower compared to protein-coding genes [14]. The expression specificity has been extended to a wide variety of physiological and pathological mechanisms like cancer development and pluripotency [15]. lncRNAs can act either proximally (in the *cis* region) or distally (in the *trans* region) interfering in the transcriptional regulation of protein-coding genes [16]. Like proteins, various lncRNAs are attributed to oncogenic or tumor-suppressive activities exerting various cellular functions [17, 18]. In addition, lncRNAs regulate gene expression at the epigenetic [19] and post-transcription levels [20]. Genome-wide association studies in cancer have disclosed that 80% of cancer-associated single-nucleotide polymorphisms (SNPs) [21] are in non-coding regions [22], including lncRNAs, suggesting that a significant portion of the genetic etiology of cancer can be related to lncRNAs. Moreover, lncRNAs are reported to be useful for disease prognosis, exemplified by the lncRNA HOTAIR [23] (HOX transcript antisense RNA), which is upregulated in acute myeloid leukemia (AML) patients.

So far, the majority of profiling studies explored the role of single lncRNAs in leukemia including AML [24], chronic lymphocytic leukemia (CLL) [25], and pediatric ALL [21, 26]. Yet a comprehensive genomic and epigenetic delineation of lncRNA deregulations in BCP-ALL subtypes and their molecular and functional insights during the evolution of the disease are lacking.

In the present study, we explored lncRNA landscapes in DUX4, Ph-like, and NH-HeH BCP-ALL subtypes and extracted novel biological and functional insights of BCP-ALL subtype-specific lncRNAs and their epigenetic activity. On the basis of RNA-seq transcriptional and DNA methylation survey of lncRNAs, we have determined 1235 subtype-specific and relapse-specific lncRNAs. Interestingly, a subset of lncRNAs were epigenetically altered. From our in-depth analyses, we have inferred the potential functions of subtype-specific lncRNAs. Overall, this work provides a most comprehensive and integrative insight that highlight the impact of lncRNAs on relevant pathways that are dysregulated in the molecular subgroups of BCP-ALL and may provide new approaches for prognosis and treatment.

## Method

### Patient samples

The patients (*n* = 45, Table 1) included in this study were selected based on the lack of fusion genes detectable by routine diagnostic workup (BCR-ABL, MLL translocations, ETV6-RUNX1) from 25 pediatric and 20 adult patients. From these patients, we had collected 40 bone marrow samples at initial diagnosis (ID) and 42 bone marrow samples at relapse (REL). All patients were treated in population-based German study trials (GMALL for adult and BFM for pediatric patients) [26]. The study was designed to include relapsed BCP-ALL patients with paired samples from diagnosis and relapse. Due to poor RNA quality, selected samples had to be excluded from further downstream analysis: these included 5 samples from ID and 3 samples from REL with insufficient quality of RNA-seq data. Out of 45 patients, 37 patients had paired samples. A written informed consent to participate in these trails according to the Declaration of Helsinki was obtained from all patients. The studies were approved by the ethics board of Charité, Berlin.

**Table 1:** The patient information of the discovery cohort

Patient and sample information			
Patients (*n* = 45) Paired (ID/REL): *n* = 37 Unpaired (ID or REL only): *n* = 8	Samples (*n* = 82) ID: 40 REL: 42		
Patients in Subtypes	Samples		ID/REL (paired/unpaired)
DUX4 (*n* = 12)	*n* = 23	ID (*n* = 12) REL (*n* = 11)	Paired (*n* = 11) Unpaired (*n* = 1; ID only)
Ph-like (*n* = 11)	*n* = 21	ID (*n* = 10) REL (*n* = 11)	Paired (*n* = 10) Unpaired (*n* = 1; REL only)
NH-HeH (*n* = 9)	*n* = 16	ID (*n* = 7) REL (*n* = 9)	Paired (*n* = 7) Unpaired (*n* = 2; REL only)
LH (*n* = 3)	*n* = 6	ID (*n* = 3) REL (*n* = 3)	Paired (*n* = 3) Unpaired (*n* = 0)
Unassigned (*n* = 10)	*n* = 16	ID (*n* = 8) REL (*n* = 8)	Paired (*n* = 6) Unpaired (*n* = 4; 2 ID/2 REL only)

The table defines the 45 patients, the number of samples from each subtype, and the number of paired and unpaired samples. We obtained 36 and 46 samples from 21 adult and 24 pediatric patients, respectively

### Overview of RNA-seq and DNA methylation array data

To generate transcriptome profiles of patient samples, mRNA was isolated by using Trizol reagent (Life Technologies, Grand Island, NY) procedure from the bone marrow mononuclear cells (MNCs) of the ID and REL samples. The paired-end RNA sequencing was performed on an Illumina HiSeq4000 platform (multiplexing) in the high-throughput sequencing core facility, German Cancer Research Center, Heidelberg, Germany. The RNA-seq was performed by using six samples per lane, which resulted in an average of 64 million mapped paired reads per sample. For methylation, genomic DNA was isolated using unstranded Allprep extraction (Qiagen, Hilden, Germany) from the bone marrow of same patients (ID and REL samples) was then hybridized onto an Illumina Infinium HumanMethylation450K. From the DNA methylation chip, we identified 60,021 probes annotated to 7190 lncRNAs.

### RNA-seq read alignment and quantitative extraction

The STAR aligner (version 2.4.0.1) [27] (2-pass alignment parameters) was used to align paired-end reads to the human genome reference. The human genome reference files used for processing RNA-seq samples were the hg19 (GRCh37) genome version for alignment and transcript annotation from GENCODE version 19 (equivalent Ensembl GRCh37). The transcriptome construction and gene-level counts for each sample were obtained using StringTie. The read count information from the files generated by StringTie was extracted using the “prepDE.py” python script provided by the StringTie [28]. We detected 84% of 13,860 lncRNAs (including 23,898 transcripts) annotated by GENCODE (V19) from our samples (FPKM > 0 for multi-exon lncRNAs and FPKM > 0 for single exonic lncRNAs) showing that our sequencing depth was good.

### Sample clustering and differential expression analysis for subtype-specific and relapse-specific lncRNAs

We performed PCA using the *prcomp* R function on 13,860 lncRNAs from RNA-seq and 60,021 CpGs on 7190 lncRNAs from DNA methylation datasets. The PCA plots were plotted using python matplotlib axes3D function. The R bioconductor package Linear Models for Microarray (*LIMMA*) *Voom* [29] was used on gene-level expression data for identifying the subtype-specific and relapse-specific differentially expressed (DE) lncRNAs. The subtype-specific DE lncRNAs were identified by implementing separate design matrix for the three subtypes (DUX4, Ph-like, and NH-HeH). For example, for DUX4 subtype, we used 23 samples from DUX4 subtype as treatment group and 59 samples from the rest of cohort as control group to perform the subtype-specific analysis. Within our cohort (82 samples from 45 patients), some patients had imbalances in matching ID or REL samples. For example, 2 pediatric and 6 adult patients had no matching ID or REL samples (Additional file 1: Table S1). LIMMA voom leveraged the sample imbalances and confounders (patient and samples) with its *duplicatecorrelation* function. We implemented *duplicatecorrelation* function, which addressed all patient effects by estimating correlations of multiple samples from the same patient while allowing us to compare across the subtypes. Additionally, we included the ID and REL time factors into the design (*makeContrasts*) to avoid the inflation of the variance due to time factor for each subtype.

The relapse-specific DE lncRNAs within each subtype were identified by analyzing DE lncRNAs ID versus REL samples within each subtype separately. The significant DE genes were assigned based on the *p* value < 0.01 and fold change of ≥ ±1.5. The lncRNAs from GENCODE version 19 (equivalent Ensembl GRCh37) were used as reference annotation. The heatmaps and correlation-based (Spearman method) hierarchical clustering of DE lncRNAs were performed on *z*-score transformed LIMMA-normalized gene expression values using the R Bioconductor package ComplexHeatmap. The validation of 1235 subtype-specific was performed by unsupervised hierarchical clustering on an independent BCP-ALL cohort (validation cohort) of 47 BCP-ALL patients.

### Differential methylation data analysis

The ID and REL samples from the same patients were assayed with the Illumina 450k methylation array. All the beta values have been normalized using the Subset-quantile Within Array Normalization (SWAN) method using Partek® Genomics Suite®. In order to detect differentially methylated regions, we used the R package bumphunter [30] using the most variant quartile of the CpG probes. Bumphunter searches for differentially methylated regions in an annotation-unbiased manner. Separate bumphunter runs have been performed for ID and REL samples for every three subtypes (DUX4, Ph-like, and NH-HeH), comparing each subtype versus the rest of the cohort on the *M* value. The cutoff was chosen individually at 0.95, the quantile used for picking the cutoff using the permutation distribution. In addition to that, 1000 resamples were performed for computing the null distribution. We associated the differentially methylated regions from three BCP-ALL subtypes using HOMER (hypergeometric optimization of motif enrichment) suite of tool with the reference file GRCh37.74, using the *-gene* parameter. The HOMER tool provided us with annotation of each probe; we separated lncRNAs from the output. The genomic regions were divided into promoter (± 2 kb from transcription start site, transcription termination site, TSS) and gene body. The gene body was defined if the CpGs were annotated in exonic, intronic, or TTS. The regions mapped to lncRNAs were then used for analysis. The significantly differentially hyper-methylated (methylation difference value ≥ 0.2; *P* value ≤ 0.05) and hypo-methylated (methylation difference value ≤ 0; *P* value ≤ 0.05) regions were used for further analysis. The intronic and intergenic differentially methylated (DM) lncRNAs were then mapped using “BedTools” with the B lymphocyte cell line “wgEncodeBroadHmmGm12878HMM.bed” in order to find the epigenetic markers. The significance of enrichment was calculated using Fisher’s exact test. The epigenetically altered lncRNAs were assigned if promoter methylated lncRNAs were differentially expressed and their DNA methylation values (log-transformed beta values) and expression values (log-transformed FPKM values) are correlated. The most significant correlations (Pearson correlations coefficient, two-tailed *P* value ≤ 0.05) were classified, later called as epigenetically altered lncRNAs.

### Functional predictions using guilt-by-association approach

In our study, we used the “guilt-by-association” [31] approach by establishing the pairwise expression correlations between DE lncRNAs (from all BCP-ALL subtypes) and its *cis* and *trans* protein-coding (PC) genes in order to predict the functions of subtype-specific lncRNAs. We determined the *cis* and *trans* PC genes of DE lncRNAs using the GREAT tool (version v3.0.0) [32]. All PC genes from GENCODE v19 annotation (*n* = 20,698) were used in the analysis. The individual *cis* and *trans* genes for each DE lncRNAs were located within a genomic window of 100 kb and greater than 100 kb, respectively. From each dataset, we then computed the pairwise expression correlation using Pearson correlation method between each lncRNAs and its *cis* and *trans* coding gene. The significantly co-expressed PC genes (Pearson correlation coefficient ≥ 0.55 and two-tailed *P* value ≤ 0.05) were further used for functional enrichment analysis using GeneSCF v1.0 [33]. The functional enrichment analysis was performed using the KEGG database with a background of all protein-coding genes from GENCODE v19 [34] (20,345). The functional terms were considered significant only if it is enriched with *P* value ≤ 0.05.

## Results

### Unique lncRNA expression profiles characterize BCP-ALL subtypes

To identify BCP-ALL subtype-specific lncRNAs, we analyzed transcriptome profiles from paired initial diagnosis (ID) and relapse (REL) samples of 25 pediatric and 20 adult BCP-ALL patients lacking known chromosomal translocations like BCR-ABL, KMT2A, and ETV6-RUNX1. Based on expression signatures of PC gene and fusion gene detection by RNA expression and DNA methylation profiles, the samples (*n* = 82) were classified into different molecular subtypes (Additional file 1: Table S1), namely, double homeobox, 4 (DUX4) (*n* = 23), Ph-like (*n* = 21), near haploid or high hyperdiploid (NH-HeH; *n* = 16), and low-hypodiploid (LH; *n* = 6) and others (*n* = 16).

**Fig. 1:**
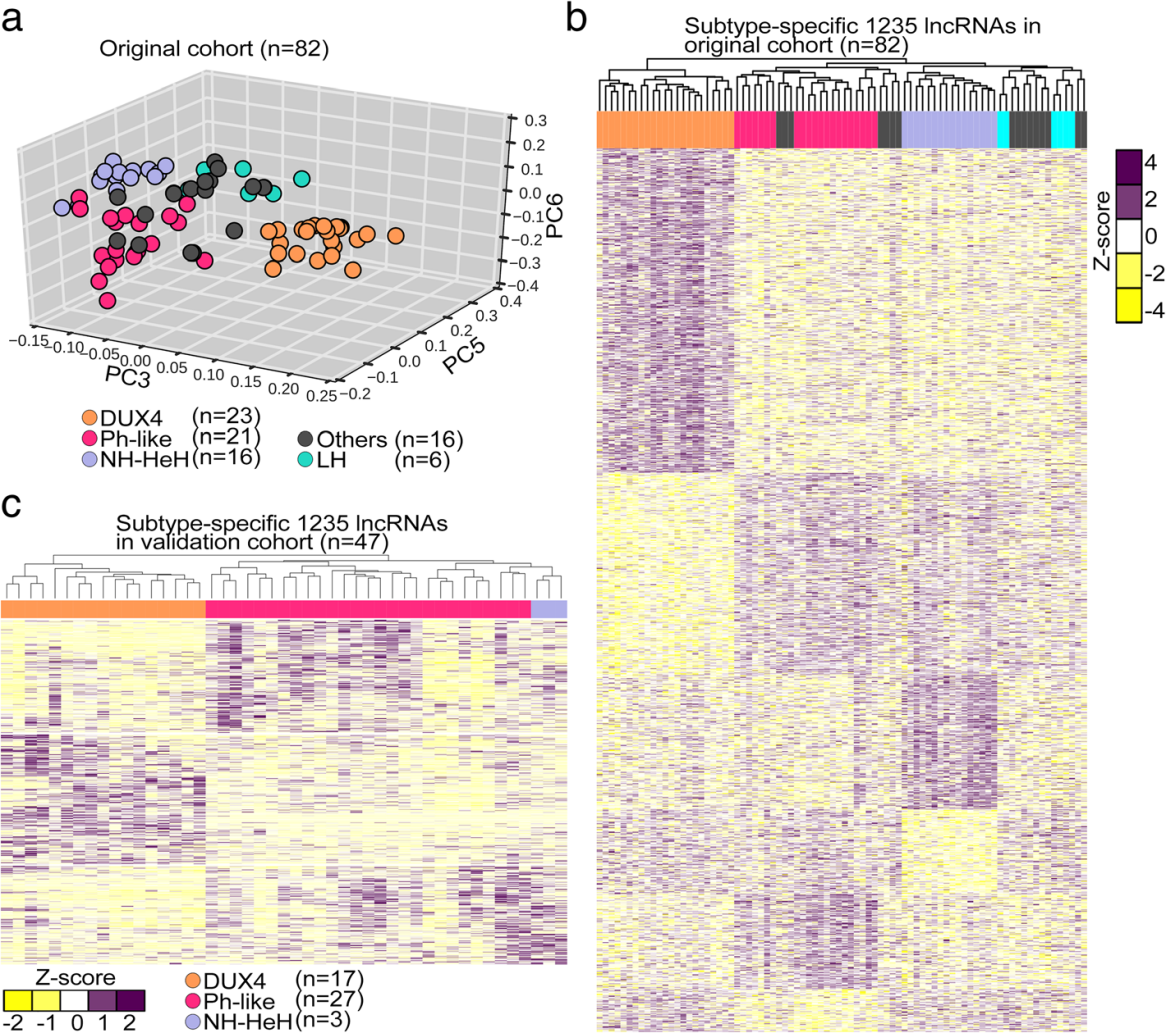
BCP-ALL subtype-specific lncRNA expression signatures on discovery and validation cohorts. **a** PCA plot constructed from expression FPKM values of lncRNAs from 82 BCP-ALL samples obtained from RNA-seq. Each point represents a BCP-ALL sample. DUX4, Ph-like, NH-HeH, LH subtype, and others are represented by orange, rose, blue, green, and gray, respectively. **b** Heatmap illustrates unsupervised hierarchical clustering on 1235 DE subtype-specific lncRNAs (absolute fold change ≥ ± 1.5, *P* value ≤ 0.01) based on *z*-score transformed LIMMA normalized expression values. The subtype-specific lncRNAs from DUX4, Ph-like, and NH-HeH subytpes displayed in the plot. **c** Unsupervised hierarchical clustering of DE subtype-specific 1235 lncRNAs on the validation cohort (*n* = 47) with z-score transformed FPKM values. The heatmap represents three distinctive clusters of DUX4, Ph-like, and NH-HeH subtypes

The unsupervised clustering using principle component analysis (PCA) on the expression (FPKM value) of 13,860 GENCODE lncRNAs revealed a distinct separation into three major BCP-ALL subtypes corresponding to DUX4, Ph-like, and NH-HeH (Fig. 1a). There was no change in subgroup classification from initial diagnosis to relapse, with all samples clustering consistently to one subgroup and no samples changed subgroup from ID to REL. This observation is in concordance to the predefined molecular classification based on PC expression signatures. In particular, samples of the DUX4 subtype showed robust separation to the remaining samples highlighting a subtype-specific lncRNA signature.

When the level of lncRNA gene expression profile across all BCP-ALL samples was compared with that of protein-coding genes, the former generally showed lower expression levels to the latter [37] (Additional file 2: Figure S1a, Additional file 1: Table S1). To unveil DE lncRNAs across these three major molecular BCP-ALL subtypes, we performed a DE analysis between the subtypes. We obtained 1235 significant DE subtype-specific lncRNAs (*P* value ≤ 0.01 and absolute fold change ≥ ± 1.5) defining signatures of three subtypes (Fig. 1b, Additional file 2: Figure S2a-c, Additional file 1: Table S1). Of these, 24 lncRNAs were commonly detected in all 3 BCP-ALL subtypes. Out of the 1235 subtype-specific lncRNAs, 23 overlapped with previously defined cancer-related lncRNAs in the lnc2cancer database [35] (Additional file 1: Table S1). For example, the oncogenic lncRNAs *PVT1* [36] and *GAS5* [37] were differentially upregulated in the DUX4 subgroup, and *EGOT* [38] was upregulated DE in Ph-like subgroup.

### Validation of the subtype-specific lncRNAs using an independent BCP-ALL cohort

To ascertain if this subtype-specific lncRNAs (*n* = 1235) could stratify the molecular subtypes of BCP-ALL samples beyond our discovery cohort (*n* = 45), we performed an unsupervised hierarchical clustering on an independent validation cohort (*n* = 47; Additional file 1: Table S1). Patients from the validation cohort included only adult patients with samples collected at ID. The result was a robust separation of DUX4, Ph-like, and NH-HeH (Fig. 1c) subtypes, which is in concordance with previous observations. The lncRNA signature classified correctly on our validation cohort with 100% sensitivity and specificity. This validation indicates the ability of our subtype-specific lncRNAs in stratification of subtypes in BCP-ALL.

### Identification and inferred functions of lncRNAs associated with molecular subtypes of BCP-ALL

As lncRNAs can exert their function by regulating protein-coding genes located at their in *cis* and/or *tran*s [39–42] regions, we performed functional enrichment analyses using guilt-by-association approach based on the correlation between neighboring (*cis*, within ±100 kb) and distally (*trans*, *> ±* 100 kb window) located protein-coding (PC) genes of the subtype-specific lncRNAs (see the “Method” section). Expression of both *cis* and *trans* PC genes showed a higher tendency towards positive correlation with the expression of the corresponding lncRNAs (Table 2).

**Table 2:** Number of BCP-ALL subtype-specific lncRNAs co-expressed with its *cis* and *trans* PC genes

Subtype	*Cis* PC genes (*n* = 929)	lncRNAs co-expressed with *cis* PC genes (*n* = 621)	*Trans* PC genes (*n* = 753)	lncRNAs co-expressed with *trans* PC genes (*n* = 552)
DUX4	669	451 (736)	492	379 (736)
Ph-like	260	170 (383)	261	173 (383)

The table represents the number of significantly (Pearson correlation coefficient ≥ 0.55, two-tailed *P* value ≤ 0.05) co-expressed subtype-specific lncRNAs with their *cis* and *trans* protein-coding genes. The numbers shown within the bracket are the total number of subtype-specific lncRNAs. *Cis* PC genes, the protein-coding genes located within the *cis* (≤ 100 kb proximity) region of the subtype-specific lncRNAs; *Trans* PC genes, the protein-coding genes located within the trans region (≥ 100 kb) of subtype-specific lncRNAs

**Fig. 2:**
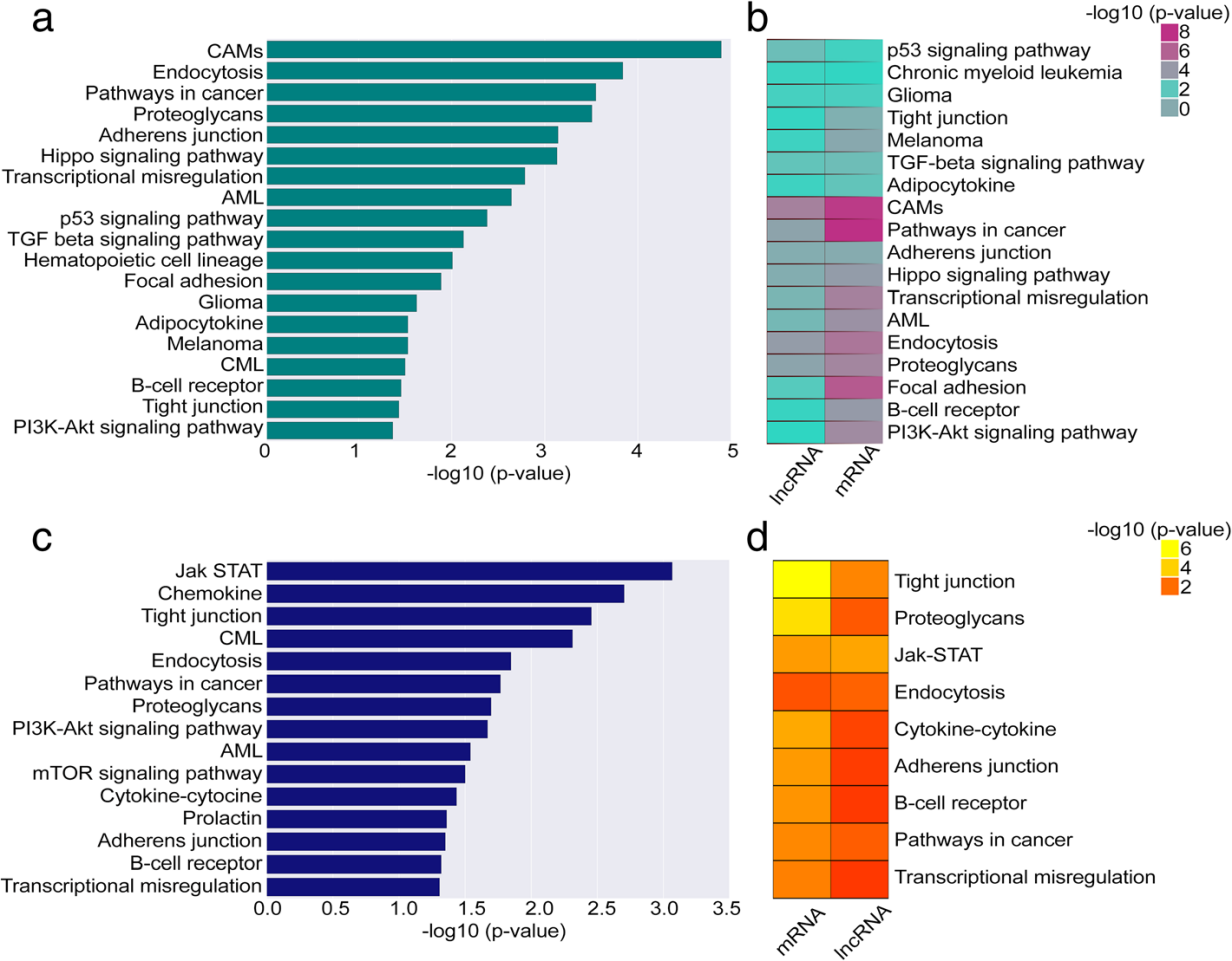
The molecular pathways of lncRNAs involved in the DUX4 and Ph-like BCP-ALL subgroups. **a** The barplot plot depicts the molecular pathway analysis from the functional enrichment analysis for nearby (≤ 100 kb proximity) *cis* protein-coding genes correlated (Pearson correlation coefficient ≥ 0.55 and two-tailed *P* value ≤ 0.05) with DE lncRNAs in the DUX4 subtype. **b** The heatmap depicts the concordance between the protein-coding and lncRNA-based predictions for DUX4 subtypes. **c** The barplot depicts the molecular pathway analysis from the functional enrichment analysis for nearby (≤ 100 kb proximity) *cis* protein-coding genes correlated (Pearson correlation coefficient ≥ 0.55 and two-tailed *P* value ≤ 0.05) with DE lncRNAs in the Ph-like subtype. **d** The heatmap depicts the overlapping pathways from both lncRNAs and protein-coding in the Ph-like subtype. The KEGG pathways or biological functions presented in the heatmaps and barplots show with *P* value ≤ 0.05 and > 2 genes involved in each pathways. The hypergeometric *P* values are obtained from GeneSCF for the pathways. CAMs cell adhesion molecules, CML chronic myeloid leukemia, AML acute myeloid leukemia

Out of these significantly co-expressed (Pearson correlation coefficient ≥ 0.55, two-tailed *P* value ≤ 0.05) *cis* protein-coding genes we identified, 58 DUX4- and 24 Ph-like-specific lncRNAs demonstrated activation of key signaling pathways involved in proliferation, apoptosis, and differentiation in leukemia (Additional file 3: Table S2). For example, in the *cis*-based co-expression analysis, we identified a strong correlation between DUX4-specific lncRNAs and genes involved in the TGF-beta, Hippo, and P53 signaling pathways (Fig. 2a, Additional file 3: Table S2). Ph-like-specific lncRNAs were correlated with genes involved in JAK-STAT, mTOR, and PIK3-AKT signaling pathways (Fig. 2c, Additional file 3: Table S2). The *trans*-based co-expression analysis revealed same vital signaling pathways in DUX4 subtype (Additional file 2: Figure S3a-b, Additional file 3: Table S2), whereas in Ph-like subtype, we identified additional signaling pathways, including P53 and mitogen-activated protein kinase (MAPK) pathways (Additional file 2: Figure S3c, Additional file 3: Table S2). The strongly co-expressed *cis* PC genes with DE lncRNAs (*n* = 32) include oncogenes such as *IL2RA* [43], *TGFB2* [44], and *CDK6* [45] activated in signaling pathways from DUX4 and Ph-like subgroups (Additional file 2: Figure S4a-d, Table 3).

**Table 3:** Subtype-specific lncRNAs and oncogenes

Subtype-specific lncRNAs	Pearson correlation coefficient	*P* value	Oncogene
RP11-347C18.3	0.56	3.25E−008	CDK6
RP11-461F16.3	0.62	5.21E−010	
RP11-96H19.1	0.62	3.89E−010	
RP11-228B15.4	0.64	7.68E−011	
MME-AS1	0.56	3.68E−008	
CTB-39G8.3	0.57	1.78E−008	
AC002454.1	0.72	2.21E−014	
RP11-582 J16.4	0.55	8.08E−008	
AC009970.1	0.64	6.23E−011	
RP11-229P13.20	0.66	1.44E−011	
LINC00114	0.57	3.06E−008	
CTB-118N6.3	0.61	9.70E−010	
SOCS2-AS1	0.62	4.94E−010	
CTD-2561B21.10	0.61	9.91E−010	
RP11-413E1.4	0.56	4.36E−008	
KB-1460A1.1	0.55	7.77E−008	
AC012309.5	0.59	4.10E−009	
RP11-37B2.1	0.59	4.76E−009	
ASB16-AS1	0.65	3.86E−011	
LINC00426	0.62	6.32E−010	
LINC01071	0.57	2.46E−008	
RP11-536K7.5	0.74	5.11E−15	IL2RA
RP11-224O19.2	0.98	1.08E−061	TGFB2
AC004837.5	0.83	6.11E−023	
RP11-251M1.1	0.79	7.39E−019	
CTD-2571L23.8	0.75	2.94E−016	
RP11-35O15.1	0.65	3.36E−011	
AC139100.3	0.58	1.00E−008	
RP11-158M2.3	0.58	1.50E−008	
RP11-672A2.5	0.56	4.68E−008	
CTD-2357A8.3	0.55	7.46E−008	
RP11-677M14.3	0.55	6.68E−008	

Positively correlating novel *cis* subtype-specific lncRNAs with oncogenes, *CDK6*, *TGFB2*, and *IL2RA* from Ph-like and DUX4 subtypes

However, there were no significant pathways identified within NH-HeH subtype. We next related the functions of DUX4 and Ph-like-specific DE lncRNAs obtained from *cis*-based analysis to those functions identified with DE PC genes. We observed an overlap of 100% (*n* = 18, Additional file 3: Table S2) of pathways from the DUX4 subtype between lncRNA-based and PC-based functional enrichment analysis (Fig. 2b). In the Ph-like subtype, we identified 60% (9 out of 15) equal pathways between DE PC-based and DE lncRNA-based functional enrichment analysis (Additional file 3: Table S2 and Fig. 2d). However, we identified Ph-like-specific lncRNAs to be strongly correlated with genes involved in key signaling pathways than Ph-like-specific protein-coding genes. For example, we identified mTOR and PI3K-AKT exclusively in the Ph-like-specific lncRNA-based pathway analysis. Together, our analyses highlight important functions of BCP-ALL subtype-specific lncRNAs whose expression correlates tightly with that of cancer-related oncogenes.

### Relapse-specific lncRNAs driving BCP-ALL progression

**Fig. 3:**
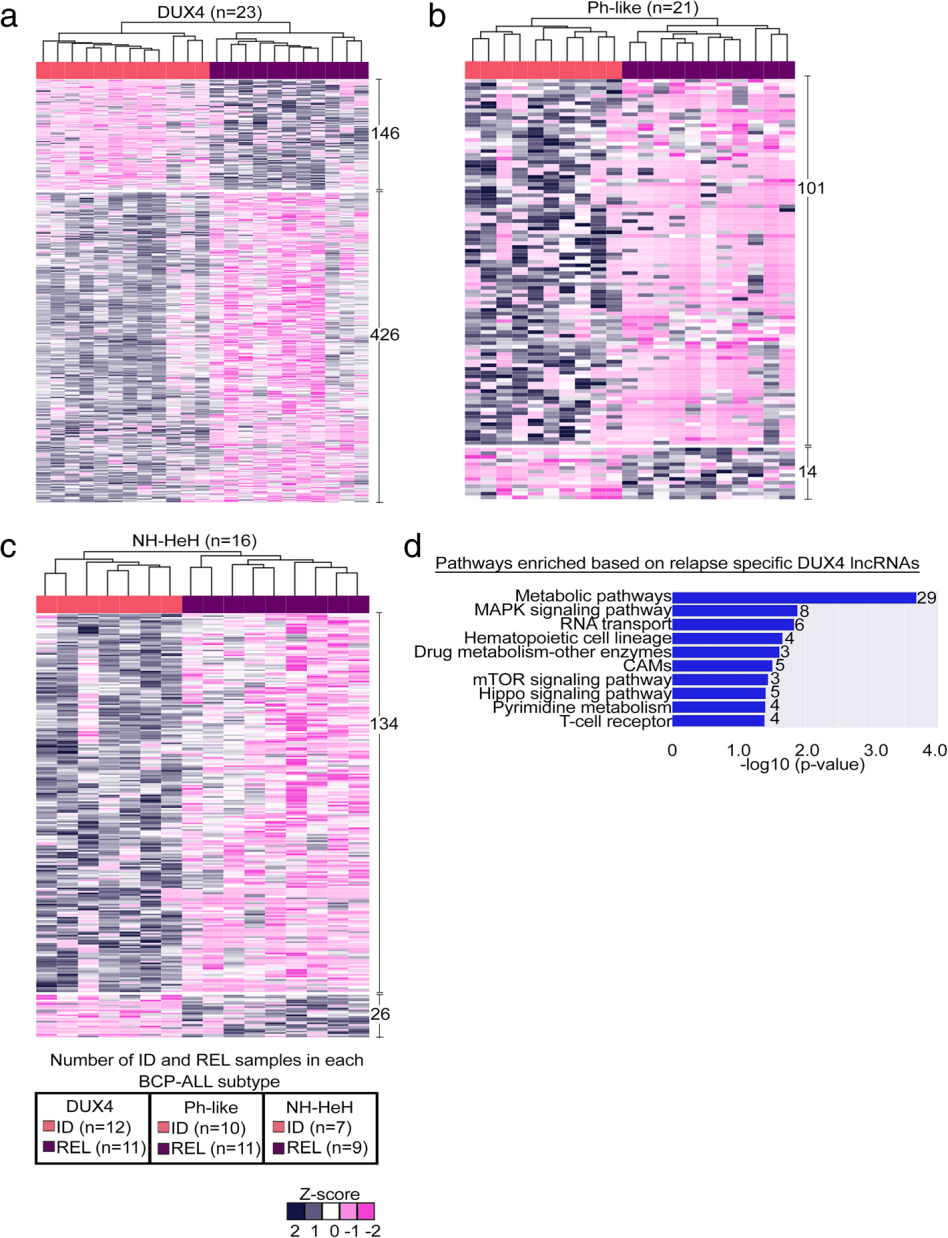
Relapse-specific DE lncRNAs from BCP-ALL subtypes. **a**–**c** Heatmap depicting the hierarchical clustering on relapse-specific DE lncRNA signature on *z*-score transformed LIMMA normalized expression values from DUX4, Ph-like, and NH-HeH subtypes. Each heatmap shows the up- and downregulated lncRNAs specific to ID and REL samples. **d** Molecular pathway analysis with the number of genes involved in each pathway from the enrichment analysis of the nearby (< 100 kb proximity) *cis* protein-coding genes correlated (Pearson correlation > 0.55 and *P* value ≤ 0.05) with relapse-specific DE lncRNAs in the DUX4 subtype. The legend box indicates the number of ID and REL samples within each group. Abbr.: CAMs; cell adhesion molecules

To gain insights into the possible role of lncRNAs driving BCP-ALL progression, we investigated dysregulation of lncRNAs at relapse. For each molecular BCP-ALL subtype, we performed a differential expression analysis of lncRNAs between ID and REL samples (Fig. 3). Nine hundred forty-seven lncRNAs (Additional file 4: Table S3) emerged as significantly DE (absolute fold change ≥ ± 1.5; *P* value ≤ 0.01) between ID and REL from the three subtypes. Around 20% (*n* = 186) of those DE lncRNAs were upregulated, and 80% were downregulated at relapse. The hierarchical clustering on relapse-specific lncRNAs within each subtype (DUX4, Ph-like, NH-HeH) identified clear separation between ID and REL (Fig. 3a–c). While majority of relapse-specific lncRNAs are novel, we identified a few previously reported onco-lncRNAs (Table 4) within our set.

**Table 4:** Previously reported lncRNAs identified as relapse-specific lncRNAs in BCP-ALL subtypes

Relapse-specific lncRNAs	Disease association
TCL6 (DUX4)	Chromosomal translocation T cell leukemia/lymphoma [49]
LINC00312 (DUX4, Ph-like, NH-HeH)	Proliferation, invasion, and migration of thyroid cancer, nasopharyngeal carcinoma [50]
miR-17-92a-1 (DUX4, Ph-like, NH-HeH)	Development, progression, and aggressiveness of colorectal cancer [51]

The differentially expressed lncRNAs between relapse (REL) and initial diagnosis (ID), from three subtypes, which were previously, reported for its disease association, selected representative examples from relapse-specific lncRNAs, which were previously identified in other diseases

The putative molecular functions of relapse-specific lncRNAs were identified using the previously described guilt-by-association approach. Relapse-specific lncRNAs within Ph-like and NH-HeH subtypes did not reveal any significant correlation with activation of pathways. In contrast, in the DUX4 subtype, we identified 56% (*n* = 321) relapse-specific lncRNAs correlated with *cis* PC genes (Additional file 4: Table S3). These DUX4 relapse-specific lncRNAs showed correlation with the PC genes involved in vital signaling pathways and metabolic pathways, including NF-kappa B-signaling pathway, cell adhesion molecule (CAMS) and metabolic pathways (number of genes involved ≥ 3 and *P* value ≤ 0.05) (Fig. 3d, Additional file 4: Table S3). These results indicate that relapse-specific markers from DUX4 subtype may be functionally engaged in metabolic and signaling pathways.

### Subtype-specific BCP-ALL lncRNAs show epigenetic alterations

**Fig. 4:**
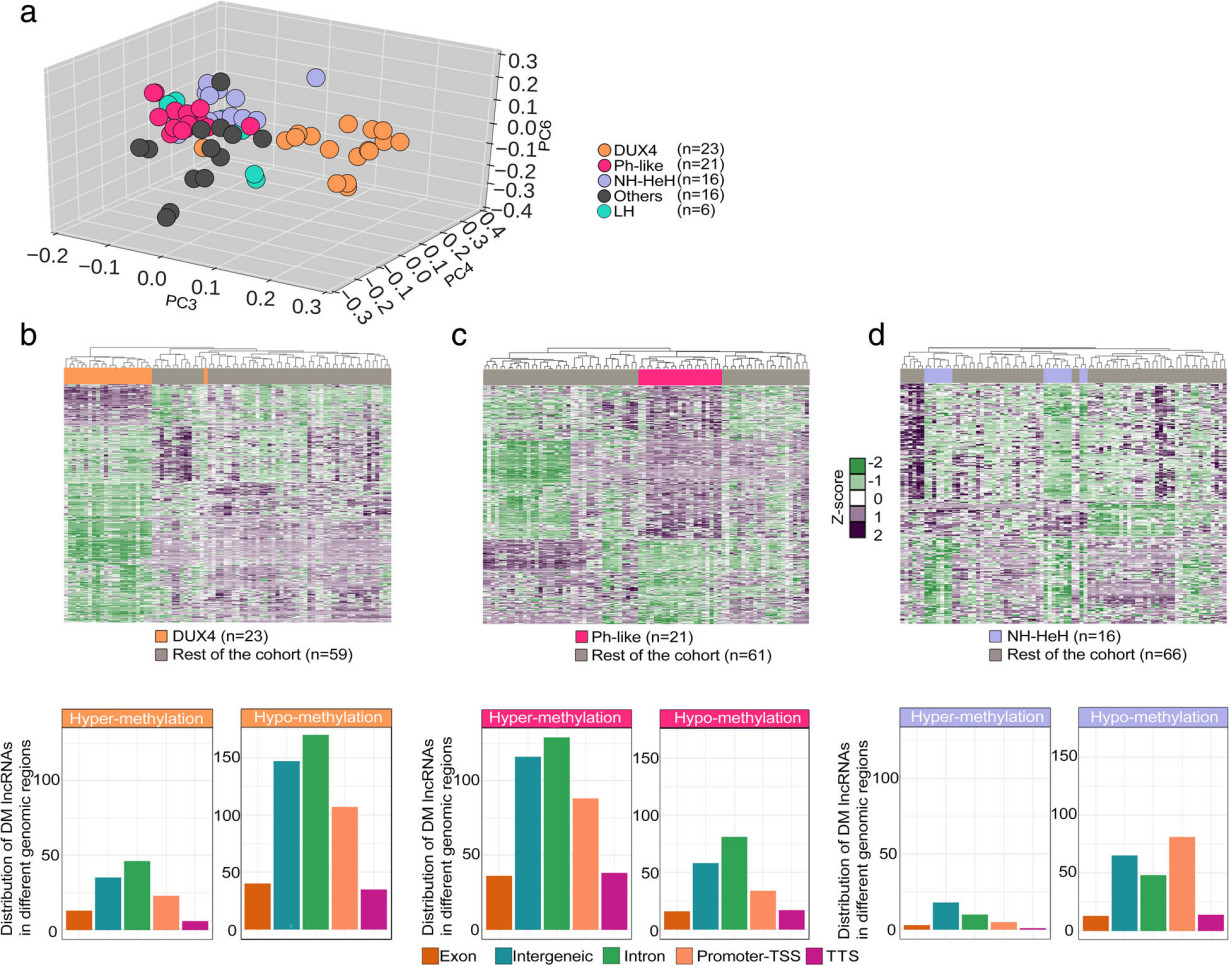
Hierarchical clustering of CGIDs associated with DM lncRNAs. **a** PCA of CpGs associated with lncRNAs on SWAN normalized β values on 82 BCP-ALL samples obtained from DNA methylation array. Each point represents a BCP-ALL sample. DUX4, Ph-like, NH-HeH, LH, and others are represented by orange, rose, blue, green, and gray, respectively. **b** The heatmap representing hierarchal clustering on 544 differentially methylated (DM) CGIDs associated with 434 lncRNAs in DUX4 subtype. In the DUX4 subtype, we identified 328 (76%) differentially hypo-methylated and 106 (25%) hyper-methylated lncRNAs. **c** The heatmap representing hierarchal clustering on 518 DM CGIDs associated with 450 lncRNAs in the Ph-like subtype. In Ph-like subtype, we observed 302 (67%) hyper-methylated lncRNAs and 148 (33%) hypo-methylated lncRNAs. **d** The heatmap representing hierarchal clustering on 295 DM CGIDs associated with 234 lncRNAs in NH-HeH subtype. In the NH-HeH subtype, we identified 200 (86%) hypo-methylated and 34 (14%) hyper-methylated lncRNAs. The heatmap is plotted using SWAN normalized beta values. The barplots below each heatmap represent the distribution of DM lncRNAs in the genome (promoter-TSS and gene body) lncRNAs from each subtype. The distribution DM promoter-TSS lncRNAs are as follows: 25%, 29%, and 39% in DUX4, Ph-like, and NH-HeH subtype, respectively

For the analysis of the methylation status of loci located at the lncRNAs genomic position in the BCP-ALL subtypes, we used DNA methylation array data (collected from Illumina 450k methylation array) from the same patients (*n* = 45) including matched ID and REL samples (*n* = 82). The distribution of DNA methylation levels of CpG sites (*n* = 60,021, Additional file 5: Table S4) associated with 7160 lncRNAs was compared with CpG sites associated with PC genes across all BCP-ALL samples. Unlike the expression levels, the distribution of DNA methylation (hypo-methylation or hyper-methylation) between lncRNAs and PC genes was similar (Additional file 2: Figure S1b). Given the robust separation of BCP-ALL subtypes on DNA methylation profile of CpGs associated with lncRNAs on the PCA analysis (Fig. 4a), we next studied the differential hypo-methylated (methylation difference value < 0; *P* value ≤ 0.05) and hyper-methylated (methylation difference value > 0.2; *P* value ≤ 0.05) CpGs associated with lncRNAs in each subtype (see the “Method” section). The hierarchical clustering of differentially methylated (DM) lncRNAs showed distinct methylation patterns for each subtype, concordant with the DE lncRNA signature (Fig. 4b–d, Additional file 5: Table S4). In the DUX4 and NH-HeH subtypes, the number of hypo-methylated lncRNAs (differential methylation value < 0, *P* value ≤ 0.05) was higher compared to the number of hyper-methylated lncRNAs. We classified the DM lncRNAs based on their genomic regions as gene body methylated and promoter-TSS methylated. In the promoter methylated lncRNAs, we identified the same trend with high degree of hypo-methylated and lower number hyper-methylated lncRNAs in DUX4 and NH-HeH subtypes. However, the Ph-like subtype has shown a higher degree of hyper-methylated DM lncRNAs than hypo-methylated DM lncRNAs. The list of subtype-specific DM lncRNAs from the three subtypes contained previously defined epigenetically altered lncRNAs from other cancer types, for example, we observed the oncogenic lncRNAs *LINC00312* [46], *PVT1*, and *TCL6* [47], which are differentially methylated in at least one of the three subtypes. Together, these data illustrate epigenetically altered list of lncRNAs in three BCP-ALL subtypes.

### Correlation between subtype-specific differentially expressed and differentially methylated lncRNAs

In order to define whether the subtype-specific promoter methylation impacts on the expression level, we compared the promoter-TSS differential CpG methylated lncRNAs (*n* = 227) with its differential expression signature. We observed 44 lncRNAs with differential methylation pattern in their promoter region accompanied by a differential expression pattern at RNA level. Out these, 23 lncRNAs harbored significant hypo-methylation and hyper-methylation pattern (Pearson correlation, two-tailed *P* value ≤ 0.05) at the promoter region (Table 5) across the three BCP-ALL subtypes.

**Table 5:** The list of significantly correlated DNA methylation and the expression of the promoter methylated lncRNAs (*n* = 23) from BCP-ALL subtypes

DM lncRNAs	Pearson correlation coefficient	*P* value	Methylation	Absolute fold change	Subtypes
AC003075.4	− 0.31	0.004	1.43	− 1.26	DUX4
AC099754.1	− 0.32	0.002	− 1.74	3.2	
AC104655.3	− 0.26	0.017	− 2.27	2.07	
CACNA1C-AS1	− 0.45	2.03E−05	1.97	− 1.62	
CTB-25B13.9	− 0.26	0.016	− 1.73	1.46	
IGF2-AS	− 0.24	0.028	− 1.33	4.95	
LINC01006	− 0.39	0.001	− 2.06	2.53	
PVT1	− 0.40	0.001	− 2.13	1.15	
RGMB-AS1	− 0.26	0.0193	− 1.48	5.96	
RP11-125B21.2	− 0.35	0.001	− 1.75	4.11	
RP11-138M12.1	− 0.70	5.21E−13	− 5.98	3.77	
RP11-367G6.3	− 0.30	0.004	1.98	− 1.63	
RP11-624M8.1	− 0.50	1.34E−06	− 3.34	4.13	
RP11-789C17.3	− 0.36	0.001	− 2.27	3.2	
SERTAD4-AS1	− 0.25	0.0232	− 1.98	1.79	
LINC01006	− 0.38	0.0003	1.44	− 1.56	Ph-like
RP11-138M12.1	− 0.70	5.21E−13	2.06	− 1.44	
RP11-305F18.1	− 0.64	5.36E−11	1.76	− 2.08	
AC099754.1	− 0.33	0.002	1.21	− 1.36	
ACVR2B-AS1	− 0.36	0.0009	2.18	− 1.75	
LINC00996	− 0.39	0.0003	− 1.56	2.11	
ERICH1-AS1	− 0.40	0.0006	− 1.82	2.21	
DIO3OS	− 0.31	0.0037	− 1.76	4.05	NH-HeH
U3	− 0.83	1.346E-22	− 2.01	2.43	

The lncRNAs are promoter differentially methylated and differentially expressed in their corresponding subtypes. DM, differentially methylated. The significance is calculated based on Pearson correlation rate and two-tailed *P* value ≤ 0.05

**Fig. 5:**
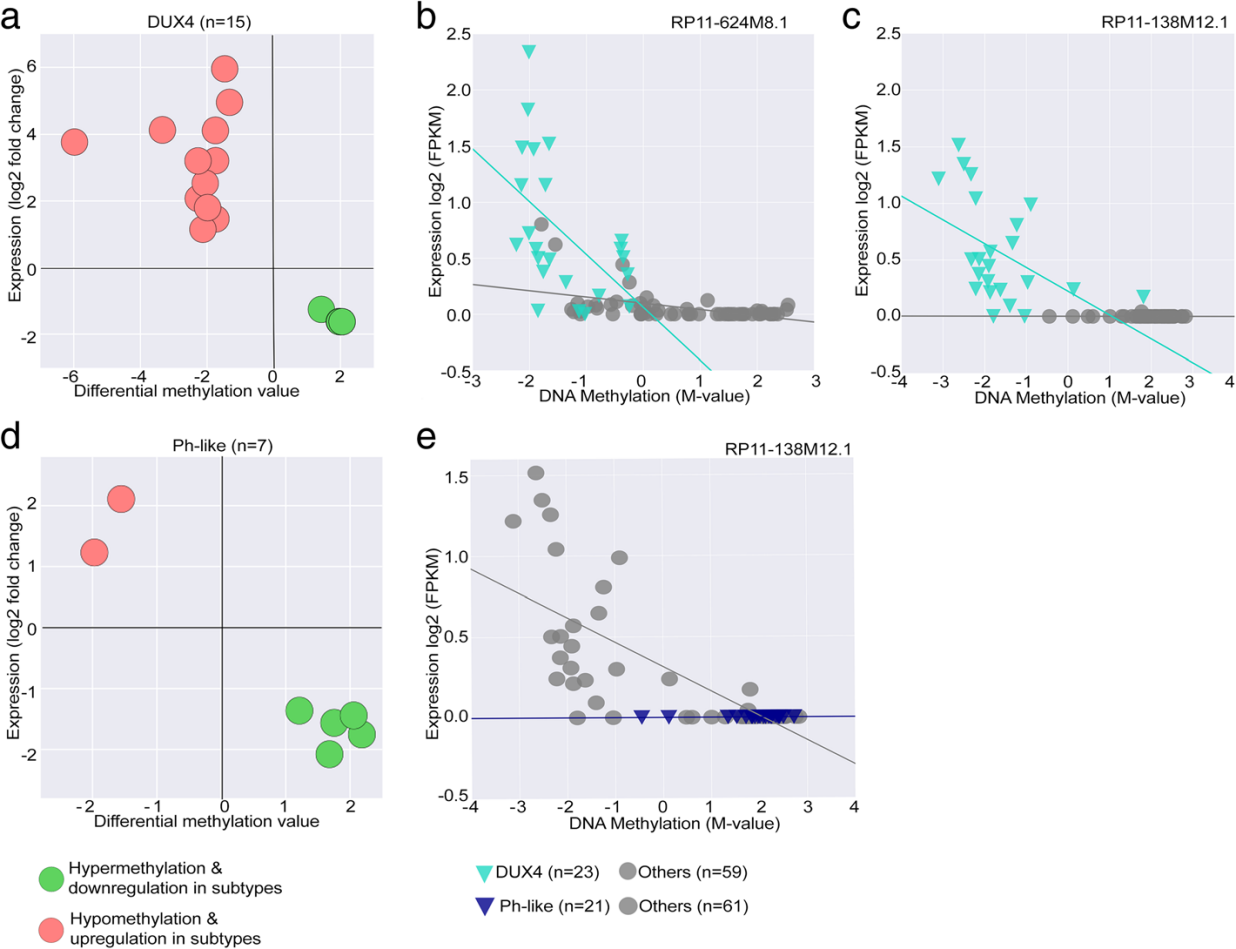
The epigenetically altered promoter methylated lncRNAs and their expression. **a** The promoter methylated lncRNAs with significant negative correlation with DE expression profile from the DUX4 subtypes. **b**, **c** Two representative examples of hypo-methylated lncRNAs with increased expression profile from DUX4 subtype. lncRNAs, *RP11-138M12.1* (Pearson correlation coefficient = − 0.69, two-tailed *P* value = 5.21E−13), *RP11-624MB.1* (Pearson correlation coefficient = −  0.50, *P* value = 1.36E−06) are examples with hypo-methylation and upregulated expression pattern with significant inverse correlation between DNA methylation and expression levels. **d** The promoter methylated lncRNAs with significant negative correlation with DE expression profile from the Ph-like subtypes. **e** A representative example of the promoter hyper-methylated lncRNA, *RP11-138M12.1* (Pearson correlation coefficient = − 0.69, two-tailed *P* value = 5.21E−13) with downregulated expression pattern, and with inverse correlation within the Ph-like subtype

Of these 23 putative epigenetically facilitated lncRNAs, 15 were related to the DUX4 subgroup (Fig. 5a) including the novel lncRNAs *R11-138M12.1* and *RP11-624M8.1*. These were significantly hypo-methylated at their promoter region and transcriptionally upregulated in the DUX4 subgroup (Pearson correlation coefficient = − 0.69; *P* value = 5.1E−13 for *R11-138 M12.1*; Pearson correlation coefficient = − 0.50; *P* value = 1.36E−06 for *RP11-624M8.1*; Fig. 5b, c). In the Ph-like subtype, we observed 7 lncRNAs with promoter methylation (Fig. 5d); interestingly, the same lncRNA *R11-138 M12.1* showed significant hyper-methylation at the promoter region and a concordant downregulation in the Ph-like subgroup (Fig. 5e). Besides these novel lncRNAs, we identified lncRNAs previously reported in the context of different cancers from our epigenetically altered results. The lncRNAs *PVT1* (Pearson correlation coefficient = − 0.40, two-tailed *P* value ≤ 0.001) and *DIO3OS* [48] (Pearson correlation coefficient = − 0.31, two-tailed *P* value = 0.0037) are examples, which we observed in the DUX4 and NH-HeH subtype with significant anti-correlation (two-tailed *P* value ≤ 0.01) to its expression level.

Around 46% (*n* = 512) of DM subtype-specific lncRNAs were localized in the intronic and intergenic genomic regions. We next aimed to investigate whether these lncRNA regions have chromatin markers encoded within their genomic location. A recent human genome-wide chromatin marker study [49] has provided a rich resource to identify chromatin markers. Genome-wide mapping of B lymphocyte cell line by searching for epigenetic markers within our DM subtype-specific intronic and intergenic regions revealed a significant number of lncRNAs (*n* = 53; Additional file 5: Table S4, Fisher extract test *P* value = 2.2E−16) with enchancer and insulator markers (Additional file 5: Table S4). Out of these, lncRNAs, *RP11-134O21.1*, *RP11-398B16.2*, *RP11-689B22.2*, *CTC-458I2.2*, and *LINC00880* were DE expressed, with a significant negative correlation between DNA methylation and the expression levels in the DUX4 subtype (Table 6). These findings suggest that epigenetic silencing and activation of promoter lncRNAs may be a mechanism that contributes to the dysregulation of expression of lncRNAs.

**Table 6:** The list of significantly correlated DNA methylation and the expression of the intronic and intergenic methylated lncRNAs (*n* = 5) from DUX4 BCP-ALL subtypes

DM lncRNAs	Absolute fold change	Methylation value	Pearson correlation rate	*P* value	Epi-markers	Biotype
RP11-134O21.1	2.54	− 1.56	− 0.63	1.9E−010	Enhancer	Intron
RP11-398B16.2	2.08	− 1.85	− 0.47	0.0007	Insulator	
RP11-689B22.2	1.52	− 3.37	− 0.47	0.008	Enhancer	
CTC-458I2.2	− 1.16	3.38	− 0.42	0.0001	Enhancer	
LINC00880	− 1.45	2.23	− 0.25	0.02	Enhancer	Intergenic

The significance is calculated based on Pearson correlation rate and two-tailed *P* value ≤ 0.05. The lncRNAs are promoter differentially methylated and differentially expressed in their corresponding subtypes. These lncRNAs are with enhancer and insulator epigenetic markers. DM, differentially methylated

## Discussion

Although previous studies have demonstrated the involvement of lncRNAs in acute leukemias [21, 25], comprehensive characterization of the transcriptome, epigenetic regulation and functional contribution of lncRNAs in distinct BCP-ALL subtypes are lacking. LncRNAs, as the novel class of functional molecules, are involved in cancer biology and have previously been reported in different molecular subtypes in various cancers. However, their role in BCP-ALL subtypes has not been investigated. Here, we unravel the lncRNA landscape using transcriptome and methylome data from 45 (adult and pediatric) relapsed BCP-ALL patients focusing on the three molecular subtypes namely DUX4, Ph-like, and NH-HeH.

Our integrated transcriptomic analyses using RNA-seq and DNA methylation bring significant novel insights and advances: they provide the most comprehensive novel datasets so far for BCP-ALL subtypes. We provide a resource of subtype-specific and relapse-specific lncRNAs and potential lncRNA functions and uncover their epigenetic alterations within the BCP-ALL subtypes. We identified 1235 DE subtype-specific lncRNAs dysregulated in at least one of the three subtypes. These 1235 DE subtype-specific lncRNAs successfully stratified subtypes in our discovery cohort, an independent validation cohort.

Another important aspect of our study is the identification of relapse-specific dysregulated lncRNAs across three BCP-ALL subtypes. A closer look into the relapse-specific lncRNA signature identified lncRNAs previously described as oncogenic including *RP11-701P16.5* [50], *SLC38A3* [51], and *LINC00312*, which are upregulated in relapsed samples within DUX4 subtype.

Importantly, apoptosis suppressor lncRNA in Myc-driven lymphomas *miR-17/92* cluster host gene (*MIR17HG*) [52] is upregulated in relapsed samples within the Ph-like subtype and downregulated in relapsed samples within DUX4 and NH-HeH subtypes. Overall, the relapse-specific lncRNAs highlight the oncogenic relevance in BCP-ALL subtypes: near haploid or high hyperdiploid (NH-HeH; *n* = 16) and low hypodiploid. Besides the oncogenic properties, lncRNAs can act as prognostic markers [53, 54] and aid at disease diagnosis and treatment. A subset of our relapse-specific lncRNAs (*n* = 61, Additional file 4: Table S3) overlaps with the prognostic markers identified from 14 Pan-Cancer datasets [42], including lung cancer-associated transcript 1 (*LUCAT1*), which is previously reported for its drug resistance in solid cancer [55]. Within the DUX4 subtype, we identified the upregulated expression of *LUCAT1* at relapse, potentially providing a novel insight into treatment resistance for BCP-ALL subtypes. Together, this illustrates the catalog of relevant lncRNAs in different subtypes of BCP-ALL as subtype-specific and relapse-specific markers with the potential of RNA-based approaches in the treatment of BCP-ALL subtypes.

The dissection of the regulatory pathways mediated by the molecular subtype-specific and relapse-specific lncRNAs revealed the activation of pivotal signaling pathways across three BCP-ALL subtypes. The functional analysis by means of the guilt-by-association approach highlights the subtype-specific and relapse-specific lncRNAs associated with activation of signaling pathways and metabolic pathways that are associated with leukemogenesis including TGF-Beta, hippo, P53, JAK-STAT, cytokine-cytokine receptor, endocytosis, mTOR, and metabolic pathways. Characterization of the lncRNAs involved in these pathways may potentially reveal novel targets in molecular therapies.

The functional insights of relapse-specific and subtype-specific lncRNAs revealed biological relevance to BCP-ALL subtypes including cell cycle functions, signal transduction, cell migration, and metabolic processes. Some of the functions predicted here corroborate previous studies emphasizing the strengths of the employed guilt-by-association. For example, lncRNA *AC002454.1*, which we associated to the PIK3-AKT pathway in Ph-like subtype, was recently reported to regulate cyclin-dependent kinase (*CDK6*) to participate in cell cycle disorder [56]. The *CDK6* gene appears to be frequently dysregulated in hematopoietic malignancies [45] and is hence attributed a critical role in tumorigenesis, also shown in ALL driven by mixed lineage leukemia (MLL) fusion proteins [57]. In Ph-like subtype, both *CDK6* and *AC002454.1* are correlated and upregulated specifically in Ph-like samples, suggesting they displayed enhancer-like functions. We identified 8 relapse-specific lncRNAs (Additional file 4: Table S3) associated with metabolic pathways in the DUX4 subtype overlapping with lncRNAs [58] reported to synergistically dysregulate metabolic pathways in multiple tumor contexts.

Besides known lncRNAs, we also identified novel lncRNAs associated with activation of key signaling pathways. For instance, in the DUX4 subtype, we identified a set of novel lncRNAs associated with TGF-beta pathway, including the antisense *RP11-224019.2*, with a significant positive correlation to the *TGFB* gene. Recently, a number of lncRNAs were documented to be associated with TGFß signaling pathway, including MEG3 regulating the TGFB2 pathway in breast cancer [40]. However, lncRNAs associated with the TGFß pathway in BCP-ALL subtypes have not been reported. The co-expression of *RP11-224019.2* and *TGFB* in DUX4 subtype may indicate their functional relatedness or regulatory relationships. In addition to that, a notable observation was a strong correlation between relapse-specific lncRNAs with genes involved in the activation of metabolic pathways in the DUX4 subtype. We identified 112 relapse-specific lncRNAs co-expressed with 29 (Additional file 4: Table S3) PC genes activated in metabolic pathways, including previously reported 8 biomarker lncRNAs. For example, we identified oncogenic lncRNA *LUCAT1* reported to be associated with poor prognosis in lung cancer [59]. However, the *LUCAT1* has not yet been reported in the BCP-ALL context. The global co-expression analysis and gene expression profiling suggest important and previously unappreciated roles of lncRNAs in the BCP-ALL subtypes. Our analyses provide important functions of subtype-specific and relapse-specific lncRNA genes whose expression correlates tightly with oncogenic coding genes.

Although we observed that subtype-specific lncRNAs and subtype-specific protein-coding genes were predicted to activate or inhibit the same pathways, some important exclusivity was observed. For instance, the signaling pathways such as the PI3K and mTOR in Ph-like subtype was enriched only in the lncRNA-based enrichment analysis, whereas these pathways did not appear to be enriched/dysregulated in the mRNA-based analysis. The PI3K and mTOR signaling pathways control proliferation, differentiation, and survival of hematopoietic cells [54]. Consistent with our results, other studies indicated the potency of lncRNAs facilitating the cancer cell growth through mTOR and PI3K signaling pathways [33, 44, 55], yet reports on BCP-ALL subtypes have been lacking so far. Considering the functional nexus between Ph-like-specific lncRNAs and the activation of pathways such as mTOR and PI3K signaling pathways, targeting those lncRNAs may be a promising novel therapeutic target for BCP-ALL subtypes.

Our work also underscores the importance of epigenetic alterations in modulating lncRNA transcriptional activities. Although previous studies [60–62] have demonstrated cross-talk between DNA methylation and transcriptional activities of lncRNAs, their role in the etiology of BCP-ALL subtypes has not been investigated. DNA methylation analyses of lncRNAs revealed that DNA methylation might underlie the differential expression of BCP-ALL subtype-specific lncRNAs. Some subtype-specific lncRNAs identified here have been reported by previous studies. For example, *SOX2-OT* (67, [63]), *LINC00312* [46], *TCL6*, and *PVT1* are onco-lncRNAs, which are promoter methylated in one of the three subtypes. The lncRNA, *PVT1*, was reported for its MYC activity [64, 65] and as oncogenic lncRNA with multiple roles in cell growth, dysfunction, and differentiation in AML [66]. Both lncRNAs, *LINC00312* and *TCL6*, have been extensively investigated on expression levels but not on the epigenetic level. The promoters of both *TCL6* and *LINC00312* were observed to be hyper-methylated with corresponding diminished expression in the DUX4 and NH-HeH samples. Notably, the DNA methylation analysis of lncRNAs revealed that DNA methylation might underlie the differential expression of subtype-specific lncRNAs.

## Conclusions

Overall, our study provides an in-depth analysis of the lncRNA transcriptome and epigenome in BCP-ALL subtypes and provides novel lncRNA markers associated with subtype and relapse specificity and with epigenetic alterations in BCP-ALL subtypes. Additionally, we also demonstrated these lncRNAs might contribute to the regulation of key signaling pathways involved in BCP-ALL. In summary, our study provides a comprehensive set of dysregulated lncRNAs from BCP-ALL subtypes derived using different integrative approaches. This subtype-specific lncRNAs and their mechanisms of action in detail might provide promising avenues for future studies to investigate key biomarkers and potential therapeutic targets in BCP-ALL subtypes.

## Additional files

Additional file 1: **Table S1.** RNA-seq data used for analysis and subtype-specific lncRNAs from three subtypes (XLSX 13450 kb)

Additional file 2: **Figure S1.** The distribution of lncRNAs and PC gene expression and DNA methylation levels across samples. **Figure S2.** BCP-ALL subtype-specific differentially expressed lncRNAs. **Figure S3.** Comparison of molecular pathways from cis and trans based analysis on subtype-specific DE lncRNAs. **Figure S4.** The subtype-specific lncRNAs co-expressed with oncogenes involved in key signaling pathways in DUX4 and Ph-like subtypes. (PDF 1510 kb)

Additional file 3: **Table S2.** The functionally involved subtype-specific lncRNAs from DUX4 and Ph-like subtypes. The *trans* and *cis* co-expresse*d* subtype-specific lncRNAs (XLSX 143 kb)

Additional file 4: **Table S3.** The relapse-specific lncRNAs from three subtypes. The lncRNAs involved in functions from DUX4 subtype (XLSX 371 kb)

Additional file 5: **Table S4.** DNA methylation array dataset. The differentially methylated lncRNAs from three subtypes. List of cis-acting epigenetically active lncRNAs. (XLSX 59994 kb)

## Abbreviations

BCP-ALL: B cell precursor acute lymphoblastic leukemia
DE: Differential expression
DM: Differential methylation
DUX4: Double homeobox, 4
JAK-STAT2: Janus kinase/signal transducers and activators of transcription
LH: Low-hypodiploid
MTOR: Mammalian target of rapamycin
NH-HeH: Near haploid or high hyperdiploid
Ph-like: Philadelphia like
PIK3-AKT: Phosphatidylinositol 3-kinase
PVT1: Plasmacytoma variant translocation 1
SOX2-OT: SOX2 overlapping transcript
TCL6: T cell leukemia/lymphoma 6
TGFß: Transforming growth factor beta
