# Supplementary material for: Long non-coding RNAs defining major subtypes of B cell precursor acute lymphoblastic leukemia
Source: J Hematol Oncol. 2019 Jan 14;12:8. doi: 10.1186/s13045-018-0692-3 (PMC6332539; doi:10.1186/s13045-018-0692-3)
Supplement: Supplementary file 2 — Figure S1. The distribution of lncRNAs and PC gene expression and DNA methylation levels across samples. Figure S2. BCP-ALL subtype-specific differentially expressed lncRNAs. Figure S3. Comparison of molecular pathways from cis and trans based analysis on subtype-specific DE lncRNAs. Figure S4. The subtype-specific lncRNAs co-expressed with oncogenes involved in key signaling pathways in DUX4 and Ph-like subtypes. (PDF 1510 kb) [file 13045_2018_692_MOESM2_ESM.pdf]

Additional figures

Fig S1: The distribution of lncRNAs and PC gene expression and DNA methylation levels across samples.

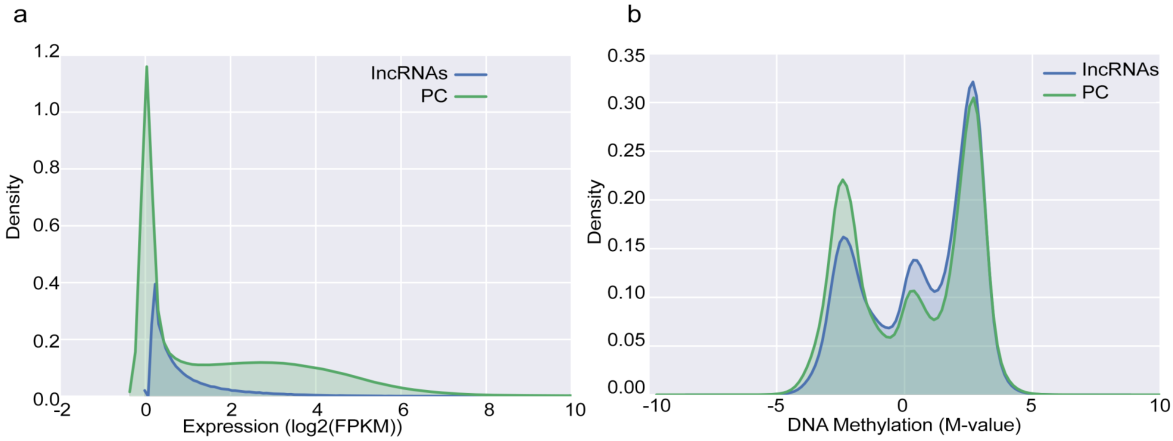

(a) The level of distribution of expression between 13460 lncRNAs and 20,135 PC genes across 82 BCP-ALL samples. (b) The level of distribution of DNA methylation rate between 60,022 CpGs probes associated with lncRNAs region and 120,000 CpGs probes associated with PC genes across 82 BCP-ALL samples.

Fig S2: BCP-ALL subtype-specific differentially expressed lncRNAs.

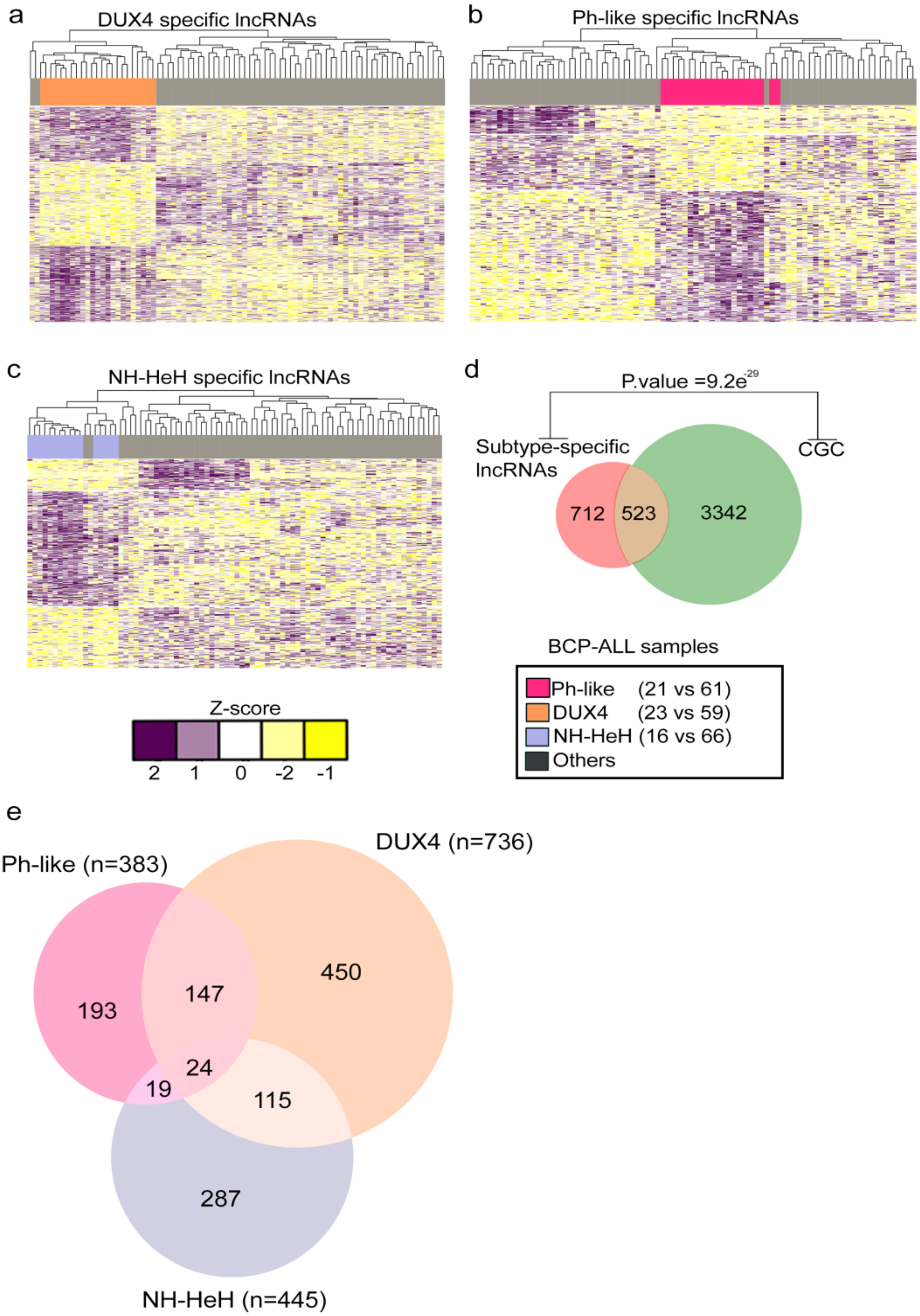

(a-c) The hierarchical clustering representing lncRNAs clustering and expression differences of the compared subtypes DUX4, Ph-like and NH-HeH; corresponding to 736, 383, and 445 subtype-specific DE lncRNAs in DUX4, Ph-like and NH-HeH subtypes, respectively. In the DUX4 subtype, 100% of samples clustered together based on the DE lncRNAs signature. The hierarchical clustering of the subtype-specific DE lncRNAs revealed that 90% (19 out of 21 samples) of Ph-like samples clustered within the predefined Ph-like subtype. For the NH-HeH subtype 69% (11 out of 16 samples) of samples correlated and clustered together using the respective DE lncRNA signature. The BCP-ALL samples box representing the number of samples within each subtype and versus (vs) the other samples used as control group in DE analysis. (d) The venn diagram illustrates the overlap between subtype-specific lncRNAs from three subtypes, showing 24 lncRNAs are to be common for all three subtypes.

Fig S3: Comparison of molecular pathways from cis and trans based analysis on subtype-specific DE lncRNAs.

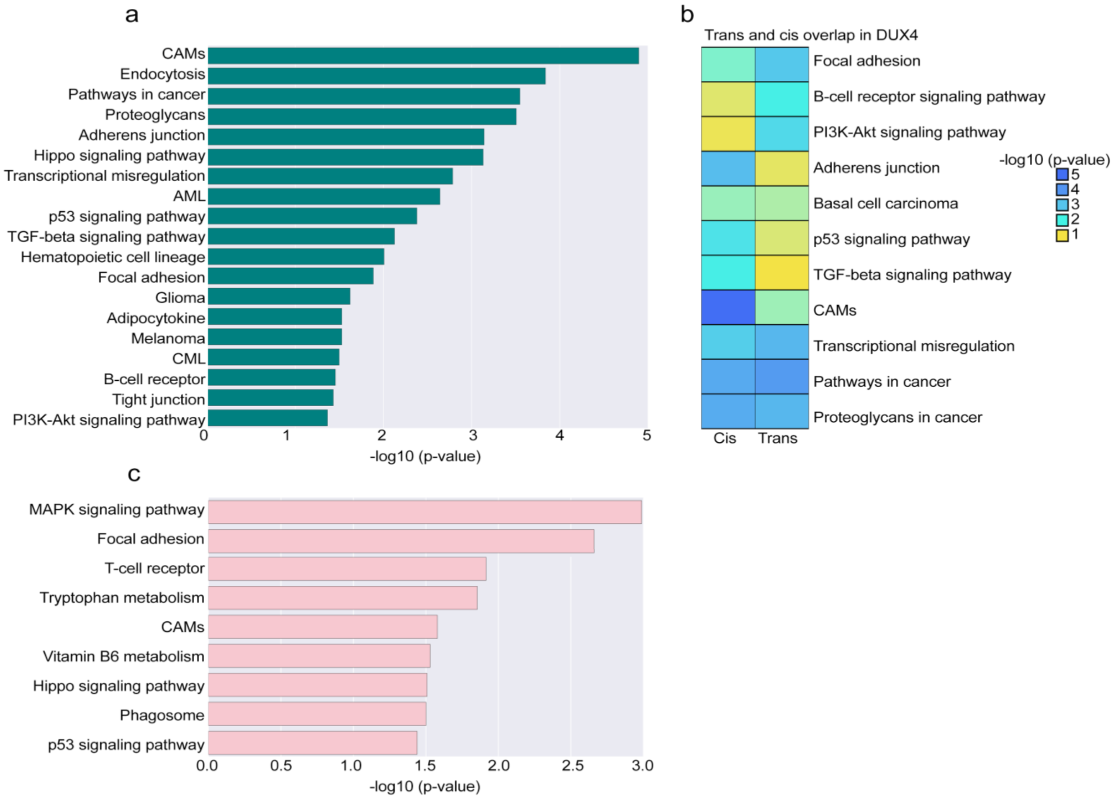

(a) Molecular pathway analysis from functional enrichment analysis on *trans* (>= 100 kb) protein-coding genes correlated (Pearson correlation coefficient >= 0.55 and two-tailed *P*-value <= 0.05) with DE lncRNAs in DUX4 subtype. (b) The molecular pathways overlapped between *cis* (< 100 kb proximity) and *trans* (> 100 kb) based functional enrichment analysis in the DUX4 subtype. (c) Molecular pathway analysis from functional enrichment analysis on *trans* (> 100 kb) protein-coding genes correlated (Pearson correlation coefficient >= 0.55 and two-tailed *P*-value <= 0.05) with DE lncRNAs in Ph-like subtype. CAMs: Cell adhesion molecules, CML: Chronic myeloid leukemia; AML: Acute myeloid leukemia.

Fig S4: The subtype-specific lncRNAs co-expressed with oncogenes involved in key signaling pathways in DUX4 and Ph-like subtypes.

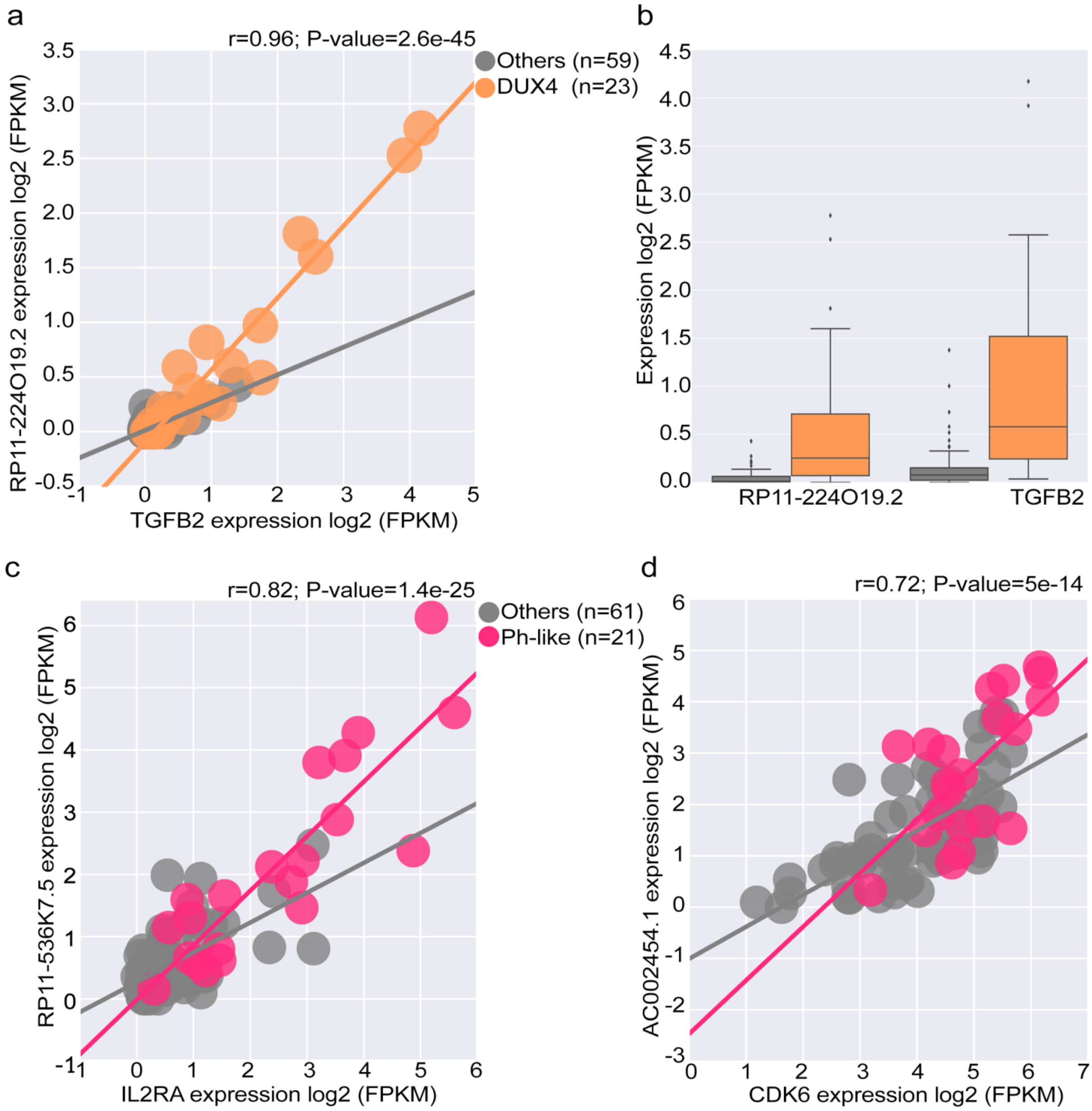

(a-b) Antisense *RP11-224O19.2* (absolute Fold change = 2.786, P-value = 9.74E-08) and its *cis* oncogene *TGFB2* (absolute Fold change = 3.84, P-value = 2.74E-10) are significantly up-regulated in DUX4 samples. (c) Antisense lncRNAs *R11-536K7.5* located at *cis* region of oncogene *IL2RA*. Expression of antisense lncRNA *RP11-536K7.5* showed significant co-expression with expression of its *cis* oncogene *IL2RA*. Both *RP11-536K7.5* (absolute Fold change = 2.79, *P*-value = 3.07E-008) and *IL2RA* (absolute Fold change = 3.11, *P*-value = 3.97e-1) are up-regulated in Ph-like samples. (d) The expression of *cis* antisense lncRNA *AC002454.1* significant co-expressed with its *cis* oncogene *CDK6* in Ph-like subtype. Both *CDK6* (absolute Fold change = 1.01, *P*-value = 0.0005) and antisense lncRNA *AC002454.1* (absolute Fold change = 1.79, *P*-value = 0.00015) are up-regulated in Ph-like samples.
